# Progress in clinical applications and mechanistic studies of the antitumor effects of Huaier granules

**DOI:** 10.3389/fphar.2026.1767429

**Published:** 2026-04-15

**Authors:** Nan Wang, Guangwei Li, Chunyuan Yan, Hongjian Zhou, Jie Xu, Jingwen Xu, Xiuhong Ren, Xin Ye

**Affiliations:** 1 Department of First Clinical Medical College, Shandong University of Traditional Chinese Medicine, Shandong Provincial Qianfoshan Hospital, Jinan, Shandong, China; 2 Shandong First Medical University, The First Affiliated Hospital of Shandong First Medical University & Shandong Provincial Qianfoshan Hospital, Jinan, Shandong, China; 3 Department of First Clinical Medical College, Shandong University of Traditional Chinese Medicine, Qingdao West Coast New Area People’s Hospital, Qingdao, Shandong, China; 4 Department of Thoracic Surgery, Central People’s Hospital of Zhanjiang, Zhanjiang, Guangdong, China; 5 Department of Radiology, People’s Hospital of Guangrao County, Guangrao, Shandong, China

**Keywords:** antitumor, clinical application, Huaier granule, integrated traditional Chinese and western medicine, mechanism

## Abstract

**Introduction:**

Huaier granules (HEG), a traditional Chinese medicinal product primarily composed of *Trametes robiniophila* Murr., have been incorporated into comprehensive treatment strategies for various cancers. When combined with conventional antitumor therapies, it has demonstrated notable adjuvant and synergistic effects, improving quality of life and prolonging survival in patients. This study aimed to systematically review the clinical efficacy, safety, and potential mechanisms of HEG as adjuvant therapy in oncology, and to critically appraise the quality of available evidence to guide clinical practice and future research.

**Methods:**

A comprehensive systematic search of PubMed, Embase, Cochrane Library, China National Knowledge Infrastructure, Wanfang, and VIP databases was conducted from inception to May 2024. Clinical studies, including randomized controlled trials (RCTs) and prospective and retrospective cohort studies, evaluating HEG combined with conventional cancer therapy were included. Preclinical studies investigating mechanisms were synthesized separately. Risk of bias was assessed using the Cochrane Risk of Bias 2.0 (RoB 2.0) tool for RCTs and the Newcastle-Ottawa Scale for observational studies. Evidence certainty was graded using a modified GRADE approach: A (high), B (moderate), C (low), and D (very low). A total of 56 studies involving 4,832 patients were included: 12 RCTs (n = 2,156), eight prospective cohorts (n = 1,089), 24 retrospective studies (n = 1,432), and 12 case series (n = 155). In hepatocellular carcinoma, HEG combined with transarterial chemoembolization (TACE) improved overall survival with a hazard ratio (HR) of 0.68 (95% confidence interval [CI], 0.52–0.89; two RCTs, moderate certainty) and progression-free survival with an HR of 0.71 (95% CI, 0.58–0.87). Similar benefits were observed when combined with chemotherapy for lung and gastric cancers (low to moderate certainty). However, most RCTs were single-center, open-label studies with heterogeneous outcome definitions. Preclinical studies suggest that immunomodulatory, antiangiogenic, and proapoptotic effects are primarily mediated by polysaccharide components (TPG-1, PS-T); however, direct clinical translation remains uncertain due to the lack of pharmacokinetic bridging data. Modern pharmacological research indicates that HEG possesses antiviral, anti-inflammatory, and immunomodulatory properties, as well as significant potential in antitumor therapy. This review comprehensively summarizes recent advances in the clinical application of HEG in antitumor therapy and explores its underlying mechanisms of action. Further in-depth research is needed to fully elucidate its mechanisms of action and ensure its safe and effective clinical use. Future clinical trials should focus on optimizing HEG application methods for various tumor types and treatment regimens to maximize its antitumor potential.

## 1 Introduction

With the rapid advancement of modern medicine and the establishment of a robust public health system, infectious and genetic diseases have been effectively controlled. Nevertheless, the incidence of malignant tumors continues to rise, making them the leading cause of death worldwide (Siegel et al., 2024; Wang et al., 2023). Malignant tumors are diseases with high mortality and low cure rates, significantly affecting patients’ physical and mental health. Their development is primarily attributed to the combined effects of external carcinogenic factors and internal changes in human constitution (Qin et al., 2022; La Torre et al., 2023). From a Western medical perspective, the pathogenesis of malignant tumors involves multiple interrelated factors, including gene mutations, clonal evolution, epigenetic alterations, dysregulation of the tumor microenvironment, immune evasion, genetic susceptibility, and environmental and lifestyle influences. These factors collectively disrupt normal cellular growth control, ultimately resulting in malignant transformation (Kiri and Ryba, 2024; Laplane and Maley, 2024; Yuan et al., 2024).

In traditional Chinese medicine (TCM), tumor formation is considered a multifactorial, multistage pathological process, mainly involving deficiency of vital Qi (Zhengqi), accumulation of pathogenic factors, Qi stagnation and blood stasis, phlegm-dampness aggregation, heat-toxin retention, and dysfunction of the Zang-fu organs (Xi et al., 2025). Conventional Western treatments—including surgery, chemotherapy, and radiotherapy—can effectively reduce tumor burden but often cause significant physical strain and severe adverse effects. TCM, alternatively, emphasizes regulating the internal milieu, reinforcing vital Qi, and eliminating pathogens, thereby inhibiting tumor progression, alleviating clinical symptoms, and improving the quality of life. However, its direct cytotoxic effect on tumors is fairly limited. The integration of TCM and Western medicine offers distinctive advantages in anticancer therapy, including.

- Improving curative outcomes: Western medicine directly targets tumor cells, whereas TCM modulates tumor-prone constitution and enhances overall resistance, resulting in higher cure rates.
- Enhancing quality of life: TCM alleviates treatment-related side effects and improves daily functioning.
- Reducing recurrence: The combined approach strengthens immune function and lowers the risk of tumor relapse.
- Prolonging survival: Integrated therapy enhances tumor control, reduces toxicity, and extends patient lifespan.
- Enabling personalized treatment: Combination strategies allow tailored therapeutic regimens based on individual conditions, thereby optimizing efficacy (Zhang X. et al., 2021; Li H. et al., 2025).

Against this background, Huaier Granules (HEG) have been widely incorporated into integrated TCM–Western antitumor therapy and have demonstrated notable clinical benefits. Despite advances in conventional therapies, combining TCM with Western medicine has gained increasing attention as a strategy to enhance therapeutic outcomes and reduce treatment-related toxicities (Zhang X. et al., 2021; Li S. et al., 2025). However, rigorous evidence supporting specific TCM interventions remains limited, necessitating a systematic appraisal of clinical efficacy and underlying mechanisms [Refer to the Frontiers Four Pillars requirements].

The primary raw material of HEG is the medicinal fungus *Trametes robiniophila* Murrill (Family: Polyporaceae; Basionym: Trametes robiniophila Murrill, Mycologia 12: 20, 1920; syn. *Phellinus robiniophila* (Murrill) Pat.) (Ji et al., 2024; Pan et al., 2019). This bracket fungus parasitizes living or dead hardwood trees, notably *Robinia pseudoacacia* L. (Fabaceae), and its dried fruiting bodies (basidiocarps) are used in TCM to clear heat, detoxify, and reinforce vital Qi (Ji et al., 2024; Pan et al., 2019). Taxonomic validity was confirmed through the Medicinal Plant Names Services (Royal Botanic Gardens, Kew; http://mpns.kew.org) and Plants of the World Online (http://www.plantsoftheworldonline.org). As a TCM substance, Huaier exerts various clinical effects, including clearing heat and detoxifying, promoting diuresis and reducing swelling, lowering blood lipids, providing anti-inflammatory and antibacterial properties, and regulating the immune system (Ji et al., 2024). However, its active pharmaceutical components are not fully understood. Currently, its main bioactive components are believed to be polysaccharide proteins, composed of heteropolysaccharides containing six monosaccharides—L-fucose, L-arabinose, D-xylose, D-mannose, D-galactose, and D-glucose—combined with 18 amino acids, including glutamic acid, aspartic acid, and glycine. The formulation of HEG involves thoroughly crushing the *T. robiniophila* Murr sclerotium and extracting it with hot water or ethanol to produce a concentrated extract, which is then mixed with appropriate amounts of sugar, dextrin, and other excipients to form granules (Pan et al., 2019). When used alone, HEG exhibits relatively limited antitumor efficacy. Therefore, in clinical practice, it is generally used as an adjunctive therapy alongside conventional antitumor treatments—including radiotherapy, chemotherapy, surgery, and palliative care—to improve overall therapeutic outcomes. HEG is a standardized commercial preparation (approved by the National Medical Product Administration: Z20000109), produced by aqueous extraction of *T. robiniophila* fruiting bodies, followed by concentration and granulation with excipients. The resulting product is a complex mixture containing polysaccharides (≥40%), proteins, and small molecular constituents, with proteoglycan TPG-1 and polysaccharide PS-T being the most studied bioactive fractions in preclinical research. However, the specific active constituents responsible for clinical efficacy remain incompletely characterized, and the relationship between these purified components and the clinically used whole extract is uncertain—a critical limitation in translating mechanistic findings into clinical practice.

## 2 Methods

### 2.1 Protocol and registration

This systematic review was conducted according to the 2020 Preferred Reporting Items for Systematic Reviews and Meta-Analyses guidelines and the GA-online best-practice recommendations for ethnopharmacological reviews.

### 2.2 Search strategy

#### 2.2.1 Information sources

The following electronic databases were systematically searched from their inception to May 2024.

- English databases: PubMed/MEDLINE (via NLM), Embase (via Elsevier), and Cochrane.
- Central Register of Controlled Trials (CENTRAL), Web of Science Core Collection
- Chinese databases: China National Knowledge Infrastructure (CNKI), Wanfang Data
- VIP Database for Chinese Technical Periodicals, SinoMed (CBM)
- Trial registries: ClinicalTrials.gov, WHO International Clinical Trials Registry
- Platform (ICTRP), Chinese Clinical Trial Registry (ChiCTR)

#### 2.2.2 Search terms

A comprehensive search was conducted in PubMed/MEDLINE, and the strategy was applied for other databases. The PubMed search strategy was as follows.

(#1) “Huaier” [Title/Abstract] OR “Trametes robiniophila” [Title/Abstract] OR “Phellinus robiniophila” [Title/Abstract] OR “Huaier granule” [Title/Abstract]
(#2) “Neoplasms” [Mesh] OR “Carcinoma” [Mesh] OR “Tumor” [Title/Abstract] OR “Cancer” [Title/Abstract] OR “Malignancy” [Title/Abstract] OR “Oncology” [Title/Abstract]
(#3) “Clinical Trial” [Publication Type] OR “Randomized Controlled Trial” [Publication Type] OR “clinical” [Title/Abstract] OR “therapeutic” [Title/Abstract] OR “treatment” [Title/Abstract] OR “efficacy” [Title/Abstract]
(#4) (#1) AND (#2) AND (#3)

The strategy for the Chinese database (CNKI example, adapted for other databases) was as follows.

SU=(‘槐耳'+'槐耳颗粒'+'槐耳浸膏'+'Trametes robiniophila'+'金克') *
SU=(‘肿瘤'+'癌'+'恶性肿瘤'+'癌症'+'癌肿') *
SU=(‘临床'+'疗效'+'随机'+'对照'+'治疗'+'应用') *
SU=(‘手术'+'化疗'+'介入'+'靶向'+'免疫'+'放疗')

### 2.3 Eligibility criteria

#### 2.3.1 Inclusion criteria

- Study design: RCTs, prospective cohort studies, retrospective cohort studies, case-control studies, and case series (≥10 patients).
- Participants: Patients with histologically or cytologically confirmed malignant tumors.
- Intervention: Huaier granules (Z20000109) as adjuvant therapy, regardless of dosage or duration.
- Comparator: Conventional cancer therapy alone (chemotherapy, surgery, interventional therapy, targeted therapy, immunotherapy, radiotherapy) or placebo.
- Outcomes: at least one of the following: overall survival (OS), progression-free survival (PFS), objective response rate, disease control rate (DCR), quality of life (QoL), or safety/toxicity profiles.

#### 2.3.2 Exclusion criteria

- Case reports (<10 patients), expert opinions, narrative reviews, and conference abstracts.
- Studies combining HEG with other TCM preparations where the specific effect of HEG could not be isolated.
- Duplicate publications (retained the complete dataset); and (La Torre et al., 2023)
- Studies with unavailable full-text or insufficient data extraction after author contact.

### 2.4 Study selection

**FIGURE 1:**
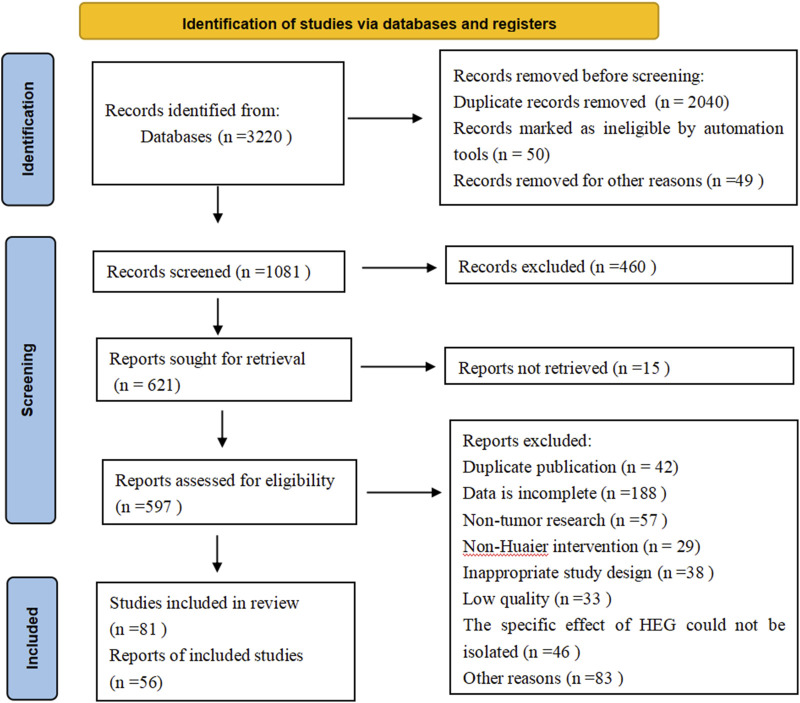
PRISMA 2020 flowchart.

Two reviewers independently screened the titles and abstracts, followed by a full-text review. Disagreements were resolved through discussion or by consulting a third reviewer. The selection process was documented using a PRISMA 2020 flow diagram. The study selection process is documented in Figure 1.

### 2.5 Quality assessment

The methodological quality of RCTs was assessed using the Cochrane Risk of Bias 2.0 (RoB 2.0) tool, which evaluated five domains of bias: (D1) bias arising from the randomization process; (D2) bias due to deviations from intended interventions; (D3) bias arising from missing outcome data; (D4) bias in measurement of the outcome; and (D5) bias in selection of the reported results.

Nonrandomized studies were assessed using the Newcastle-Ottawa Scale, which evaluates methodological quality across three domains: selection, comparability, and outcome.

### 2.6 Evidence grading

As shown in Table 1, we applied a modified GRADE approach adapted for ethnopharmacological reviews.

**TABLE 1:** Application of a modified GRADE approach adapted for ethnopharmacological reviews.

Grade	Criteria	Interpretation
A (high)	Multicenter RCTs, low risk of bias, consistent results, direct evidence	High confidence in the effect estimate
B (moderate)	Single-center RCTs or high-quality prospective cohorts with moderate bias risk	Moderate confidence; true effect likely close to the estimate
C (low)	Retrospective cohorts or case-control studies, risk of confounding	Limited confidence; the true effect may differ substantially
D (very low)	Case series, expert opinion, major methodological limitations	Very uncertain estimate

RCT, randomized controlled trial; GRADE, grading of recommendations assessment, Development and Evaluation.

## 3 Literature review

### 3.1 Clinical application of HEG in integrated traditional Chinese and Western medicine antitumor therapy

#### 3.1.1 Combined application of HEG and chemotherapy drugs

Chemotherapy, a cornerstone of Western antitumor therapy, remains one of the most important and dominant approaches for many cancers. However, its effectiveness is often limited by side effects and dose-related toxicities. Studies have shown that HEG can mitigate the adverse effects of chemotherapeutic agents and improve patient tolerance. Qu et al. investigated the safety and immunomodulatory effects of HEG combined with paclitaxel and platinum-based chemotherapy in older patients with breast cancer and found that the combination group exhibited greater clinical safety, improved QoL, and enhanced immune function compared to the control group (Qu et al., 2020). Furthermore, Huaier has been shown to reverse chemotherapy resistance in non-small cell lung cancer (NSCLC) through a distinct molecular mechanism. In cisplatin-resistant NSCLC, Huaier suppresses IL-8 expression by inhibiting the transcriptional activities of NF-κB and AP-1. Kaempferol, a key bioactive component of Huaier, targets the JNK/JUN signaling axis, thereby inhibiting IL-8 expression, reducing cisplatin resistance, and suppressing cancer cell stemness (Jin et al., 2024). Similarly, in pancreatic cancer, HEG inhibits FoxM1expression and nuclear translocation, reducing gemcitabine-induced stemness and enhancing chemosensitivity both *in vitro* and *in vivo*. These findings collectively underscore the potential of HEG as an adjunctive therapy to overcome chemoresistance by modulating key signaling pathways and transcription factors associated with cancer stemness (Wang Q. et al., 2024). Xie et al. also reported that combination therapy improved immune parameters—including T lymphocyte subsets (CD3^+^, CD4^+^, CD4+/CD8+) and NK cells—QoL, and survival rates, while reducing the incidence of bone marrow suppression, gastrointestinal reactions, and tumor recurrence (Xie et al., 2022). Similar conclusions have been reported in studies on breast, gastric, liver, and lung cancers (Ming-qian et al., 2017; Ming et al., 2020; Fu et al., 2019; Hou et al., 2020).

#### 3.1.2 Combined application of HEG and surgery

Surgical resection remains the preferred treatment for early-stage malignant tumors. Advances in technology have enabled surgery combined with systemic antitumor therapies to show significant efficacy in some intermediate- and advanced-stage cancers. As a TCM with inherent antitumor properties, HEG has demonstrated promising results when combined with surgical treatment. Lei et al. evaluated the effects of HEG as adjuvant therapy on immune function and postoperative survival in patients with triple-negative breast cancer. In a case-control study of 95 patients who underwent modified radical mastectomy followed by adjuvant chemotherapy, the combination group exhibited better Karnofsky Performance Status (KPS) scores, longer disease-free survival (DFS), and improved immune-related indicators compared to the control group, indicating that HEG adjuvant therapy can improve postoperative QoL, enhance immune function, and prolong survival (Shan et al., 2016). Li et al. investigated the use of HEG in patients after radical liver cancer resection and monitored for recurrence and metastasis; they found that the HEG group showed significant improvements in both clinical efficacy and QoL compared to the control group (Jie, 2022). A retrospective analysis of 128 patients who underwent curative resection for colorectal liver metastases revealed that postoperative adjuvant therapy with HEG combined with chemotherapy significantly improved both 3- and 5-year OS (83.5% vs. 65.3%; 60.7% vs. 48.2%, p = 0.015) and recurrence RFS (77.8% vs. 59.7%; 53.5% vs. 35.7%, p = 0.003) (Huangliang et al., 2020). Multivariate analysis further identified HEG therapy as an independent prognostic factor for improved OS. In a multicenter RCT (n = 1,044) conducted by Chen et al., adjuvant use of HEG after radical resection of hepatocellular carcinoma (HCC) significantly improved patient outcomes, with the HEG group exhibiting a median recurrence-free survival (RFS) of 75.5 weeks (HR, 0.67; p = 0.0001), an OS rate of 95.19% (p = 0.0207), and a significantly lower extrahepatic recurrence rate (p = 0.0018) compared to the control group (Chen Q. et al., 2018). Similarly, in the postoperative adjuvant treatment of triple-negative breast cancer, HEG showed efficacy in stage III patients. The experimental group achieved 5-year DFS and OS rates of 81.3% and 87.5%, respectively, which were significantly higher than those of the control group (53.8% and 65.4%, respectively); additionally, patients undergoing prolonged HEG therapy for 18 months had a significantly lower disease progression rate (Wang M. et al., 2019). Collectively, adjuvant therapy with Huaier granule has been shown in a large-scale randomized controlled trial to significantly prolong recurrence-free survival and reduce extrahepatic recurrence in patients with hepatocellular carcinoma after curative liver resection, supporting its clinical value as an adjunct to surgical treatment for liver cancer (Zhang W. et al., 2021). However, further large-scale studies are warranted to elucidate its underlying mechanisms and optimize clinical application.

#### 3.1.3 Combined application of HEG and interventional therapy

Interventional therapy is a crucial minimally invasive method for treating malignant tumors. It involves precise targeting of tumors via natural body cavities or percutaneous puncture to achieve localized tumor control, reduce tumor volume, and improve therapeutic outcomes. Despite its widespread use, interventional therapy alone has limitations, such as incomplete tumor eradication, local recurrence, and significant treatment-related adverse effects (Elsayed and Solomon, 2023). To address these challenges, integrated strategies combining traditional Chinese and Western medicine are being actively explored to enhance both efficacy and safety. Clinical studies suggest that HEG can improve outcomes when used in conjunction with interventional regimens. For example, in patients with hepatitis B virus-negative HCC, combining HEG and pirarubicin-based interventional therapy resulted in better clinical outcomes and fewer adverse reactions (Wang et al., 2018). A network meta-analysis of 33 trials further indicated that HEG significantly improved treatment response rates, OS (at 0.5, 1, and 2 years), and immune function in patients with gastrointestinal cancer, without increasing toxicity (Ma et al., 2018). The most notable benefits were observed when HEG was combined with TACE, ^125^I seed implantation, or platinum/adriamycin-based chemotherapy. Several studies specifically support the adjunctive use of HEG in ablation-based therapies. Xie et al. reported that in HCC patients meeting the Milan criteria and undergoing microwave ablation, adjuvant HEG significantly prolonged PFS, OS, and survival from extrahepatic metastasis, with enhanced benefits observed after 6 months of treatment (Xie et al., 2024). Similarly, another study found that integrated HEG therapy significantly reduced recurrence and extrahepatic metastasis in early-stage HCC patients undergoing thermal ablation, particularly in those aged <65 years with a single tumor (≤3 cm). Extended PFS was observed with continuous HEG administration beyond 2 years, and the treatment demonstrated a favorable safety profile (Wang Z. et al., 2021). Furthermore, a network meta-analysis by Zhang et al. showed that combining HEG with TACE significantly improved KPS scores in patients with primary liver cancer compared to TACE alone, with the difference being statistically significant (Rong-rong et al., 2021). Additional studies have consistently confirmed that HEG, as an adjuvant therapy, enhances the clinical efficacy of thermal ablation for HCC (Ye-fu, 2008; Kong et al., 2023; Xun et al., 2025).

#### 3.1.4 Combined application of HEG and targeted therapy

Targeted therapy is a cornerstone of precision oncology, selectively acting on specific molecular pathways in tumor cells to minimize damage to healthy tissue and improve treatment efficacy and QoL. However, its benefits are often limited by low response rates, drug resistance, and tumor recurrence (Liu, 2016). The integration of targeted agents with TCM, such as HEG, presents a promising strategy to overcome these limitations through potential synergistic antitumor effects and immunomodulation. HEG has demonstrated complementary benefits in advanced HCC, where sorafenib is the first-line targeted treatment but has limited efficacy and tolerability. Preclinical studies indicate that HEG promotes autophagy to enhance sorafenib sensitivity through mTOR-mediated pathways (Zhang et al., 2022) and inhibits metastasis in sorafenib-resistant HCC via the miR-31-5p/SPRED1 axis (Wang L. et al., 2024). Clinically, adding HEG to targeted and immunotherapy regimens significantly prolongs PFS and improves QoL in previously untreated patients with unresectable HCC (Li H. et al., 2025). Beyond HCC, Huaier extract also enhances the efficacy of tyrosine kinase inhibitors (TKIs) in high-risk hematologic cancers. For example, in Ik6+ Ph + acute lymphoblastic leukemia, it synergizes with imatinib to suppress proliferation, induce apoptosis, and inhibit BCR-ABL signaling (Qu et al., 2019). Case reports also indicate its potential utility in multimodal treatments for rare tumors, such as hepatic epithelioid hemangioendothelioma (Xu et al., 2020). Additionally, multiple studies confirm that HEG acts synergistically with targeted therapies in HCC, lung cancer, and other malignancies, highlighting its role in enhancing therapeutic response and survival outcomes (Lin-lin et al., 2022).

#### 3.1.5 Combined application of HEG and immunotherapy

Immunotherapy has become a pivotal modality in oncology, but its effectiveness is often limited by suboptimal response rates and immune-related adverse events. HEG, with its documented antitumor, immunomodulatory, and anti-inflammatory properties, represents a rational combinatorial partner that may enhance host immunity and reduce treatment-related toxicity. Mechanistic studies indicate that HEG modulates innate and adaptive immune responses. Its active proteoglycan component, TPG-1, activates TLR4-NF-κB/MAPK signaling, enhancing macrophage production of nitric oxide and proinflammatory cytokines that promote leukocyte infiltration and inhibit tumor growth (Yang et al., 2019). In colorectal cancer, HEG upregulate MHC class I expression via the STAT1 pathway, leading to increased CD8^+^ T-cell infiltration and cytotoxicity, as well as regulatory T-cell depletion, thereby reversing immunosuppressive microenvironments (Chen et al., 2025). HEG also synergizes effectively with anti-PD-1 therapy, enhancing T cell-mediated tumor killing in patient-derived organoids and murine models (Xu et al., 2025). In triple-negative breast cancer, HEG counteracts immunosuppression by inhibiting TGF-β/SMAD-dependent formation of myofibroblastic cancer-associated fibroblasts, converting immunologically “cold” tumors into “hot” tumors, and enhancing the response to PD-1 blockade (Li C. et al., 2024). Clinical observations support these findings. Yin et al. analyzed 67 PD-L1-positive advanced breast cancer patients receiving PD-1 inhibitors plus HEG and found that the combination group exhibited better DCR, KPS, and T-cell immune function, along with prolonged PFS and OS (Yu-lai et al., 2024). Another study in H22 tumor-bearing mice showed that HEG combined with anti-PD-L1 antibodies promoted CD8^+^ T-cell infiltration, upregulated PD-L1 expression, downregulated VEGFA, and produced significant antitumor effects with a favorable safety profile (Li H. et al., 2024). These results establish HEG as a multifaceted immunoadjuvant that enhances responses to immune checkpoint inhibitors across various cancer types.

#### 3.1.6 Application of HEG in radiotherapy

**FIGURE 2:**
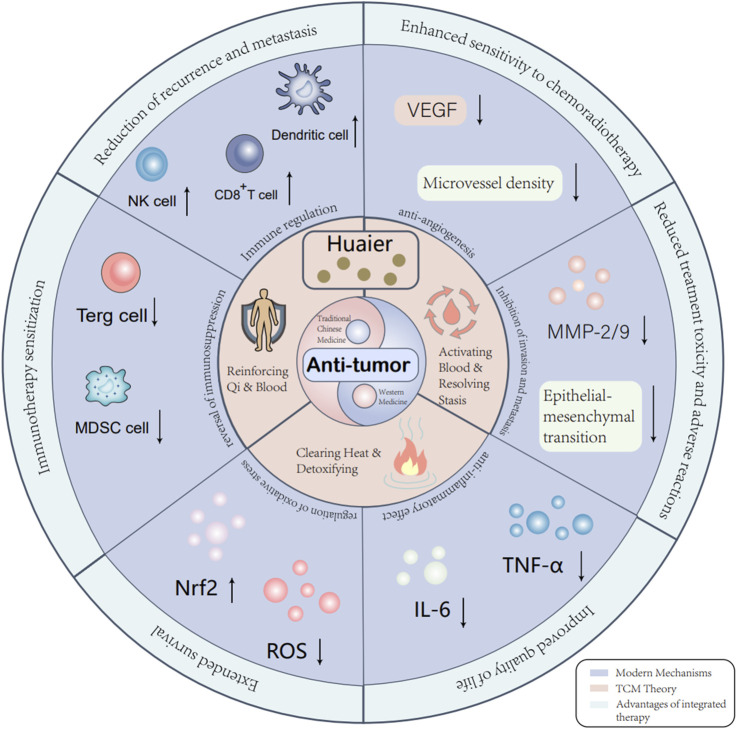
The advantages of integrating traditional Chinese medicine and Western medicine.

Radiotherapy is a fundamental local cancer treatment that can modestly stimulate antitumor immunity but often lacks systemic efficacy. Theoretically, HEG may complement radiotherapy through its immunomodulatory and anti-inflammatory effects by potentially improving immune function, increasing radiosensitivity, reducing localized side effects, and enhancing patient tolerance. Preclinical evidence indicates that HEG functions as a radiosensitizer in breast cancer models by inducing G0/G1 cell cycle arrest and inhibiting DNA repair through downregulation of RAD51 and sustained γ-H2AX foci after irradiation (Ding et al., 2016). Although a network meta-analysis did not identify significant survival benefits with HEG monotherapy in resected HCC, its mechanistic support for radiation synergy underscores its potential in combination regimens (Ye et al., 2023). Clinical studies have begun to validate these insights. Zhang et al. evaluated 60 patients with nasopharyngeal carcinoma undergoing concurrent chemoradiotherapy and found that adding HEG significantly improved T-cell immune function and reduced fatigue, nausea, vomiting, and pain scores compared to controls (Cai-you et al., 2020). Other reports also suggest that HEG improves prognosis and immune function in patients with breast, nasopharyngeal, esophageal, and lung cancers undergoing concurrent chemoradiotherapy (Lan-Ning et al., 2003). These collective findings support the potential of HEG as a beneficial adjunct to radiotherapy in various malignancies, warranting further clinical investigation. Tables 2, 3 integrate clinical and key supporting preclinical evidence across various combination modalities, providing a multilevel overview of the existing research on HEG. The advantages of integrating traditional Chinese medicine and Western medicine are Chinese medicine and Western medicine are conceptually illustrated in Figure 2.

**TABLE 2:** Summary of clinical studies and trials on integrated traditional Chinese and Western medicine antitumor therapy: clinical evidence.

Species	Sample size (T/C)	Cancer type	Intervention (T)	Intervention (C)	Intervention time	Outcome and adverse events	Stage	Primary endpoint	References
Human	65 (35/30)	Breast cancer (elderly)	Huaier granule + TC (cyclophosphamide 0.6 g/m^2^ + docetaxel 75 mg/m^2^)	TC (cyclophosphamide 0.6 g/m^2^ + docetaxel 75 mg/m^2^)	2 years	Improved patients’ quality of life and enhanced key immune markers (CD4^+^ and NK cells) with a good safety profile	Postoperative adjuvant	KPS score: CD4^+^, CD4+/CD8+, NK cells	Qu et al. (2020)
Human	1,049 (539/510)	Gastric cancer	Huaier granule + various postoperative adjuvant chemotherapy regimens (FEC/TC/TAC/TP/PC/ECT/VE, etc.)	Postoperative adjuvant chemotherapy alone	6 weeks to 2 years (variable)	Reduced bone marrow suppression, gastrointestinal reactions, and recurrence rates	Postoperative adjuvant	CD3^+^, CD4^+^, CD8^+^, CD4+/CD8+, NK cells; KPS score; quality of life; myelosuppression; gastrointestinal reaction; 2/3/5-year survival rate; recurrence rate	Xie et al. (2022)
Human	90 (45/45)	Breast cancer	Huaier granule + FEC (cyclophosphamide 0.6 g/m^2^ + epirubicin 100 mg/m^2^ + 5-fu 0.6 g/m^2^)	FEC (cyclophosphamide 0.6 g/m^2^ + epirubicin 100 mg/m^2^ + 5-fu 0.6 g/m^2^)	21 days/cycle	Reduced serum tumor markers, improved the functional status, and decreased the incidence of emotional symptoms	Postoperative (stage I-IV)	NK cells, CD3^+^, CD4^+^, and CD4+/CD8+ ratio; KPS score	Ming-qian et al. (2017)
Human	100 (50/50)	Advanced gastric cancer	Huaier granule + paclitaxel (85 mg/m^2^) + oxaliplatin (120 mg/m^2^)	Paclitaxel (85 mg/m^2^) + oxaliplatin (120 mg/m^2^)	6 cycles (7 days/cycle)	Improved quality of life	Advanced (stage III-IV)	CEA, CA724, CA199; CD3^+^, CD4^+^, CD8^+^, CD4+/CD8+; SF-36 quality of life; adverse reactions	Ming et al. (2020)
Human	95 (56/39)	Triple-negative breast cancer	Huaier granule + postoperative chemotherapy (FEC/AC/TAC/ECT + huaier)	Postoperative chemotherapy alone	1.5 years	Improved immune function, increased quality of life, and prolonged DFS	Postoperative adjuvant	2-year recurrence rate; CD3^+^, CD4^+^, NK cells; quality of life	Shan et al. (2016)
Human	Not specified	Hepatocellular carcinoma	Huaier granule	Surgery alone or a placebo	Not specified	Improved clinical efficacy and quality of life	Postoperative	Recurrence, metastasis, and survival rates	Jie (2022)
Human	84 (42/42)	Colorectal liver metastasis	Huaier granule + postoperative chemotherapy (FOLFOX4/XELOX)	Postoperative chemotherapy alone	Median 24 months (range 12–36 months)	Improved 3- and 5-year OS and RFS	Postoperative (after curative resection)	1-year, 2-year, and 3-year RFS-fre; OS	Huangliang et al. (2020)
Human	665 (334/331)	Hepatocellular carcinoma	Huaier granule (20g, TID)	Placebo	104 weeks (2 years)	Improved median recurrence-free survival and reduced extrahepatic recurrence	Postoperative (after curative resection)	RFS and OS	Chen et al. (2018a)
Human	96 (48/48)	Triple-negative breast cancer	Huaier granule (20g, TID) + postoperative chemotherapy	Postoperative chemotherapy alone	6 months	Improved 5-year DFS and OS rates	Postoperative adjuvant	2-year DFS; CD3^+^, CD4^+^, CD8^+^, NK cells; quality of life; adverse events	Wang et al. (2019a)
Human	120 (60/60)	HBV DNA-negative hepatitis B (with liver cancer background)	Huaier granule + interventional treatment (TACE/RFA)	Interventional treatment alone	6 months	Results in better clinical outcomes and fewer adverse reactions	Not specified	ALT, AST, TBIL, ALB; HBV DNA-negative conversion rate; CD3^+^, CD4^+^, CD8^+^, NK cells; KPS score	Zhang et al. (2021b)
Human	Multiple studies (network meta-analysis)	Gastrointestinal cancer	Huaier polysaccharides + adjuvant chemotherapy/therapy	Chemotherapy/therapy alone or placebo	Varied	Improved treatment response rates and overall survival	Adjuvant	Objective response rate, disease control rate, quality of life, immune function, and adverse events	Ma et al. (2018)
Human	254 (127/127 (after PSM))	Hepatocellular carcinoma (within the Milan criteria)	Huaier granule (long-term oral administration) + microwave ablation	Microwave ablation alone	Median 36.5 months (range 12–72 months)	Prolonged PFS, OS, and extrahepatic metastasis survival	Early-stage HCC (within the Milan criteria)	OS, RFS, PFS	Xie et al. (2024)
Human	316 (158/158 (matched cohort))	Early-stage hepatocellular carcinoma	Huaier granule + thermal ablation	Thermal ablation alone	Median 38.6 months	Reduced recurrence and extrahepatic metastasis rates, especially among those younger than 65 years with a single tumor ≤3 cm in diameter	Early-stage HCC	1-year, 2-year, 3-year, and 5-year RFS; OS	Wang et al. (2021a)
Human	Multiple studies	Primary liver cancer	Various oral Chinese patent medicines (including huaier granule) + conventional treatment	Conventional treatment alone or a placebo	Varied	Improved the KPS score	Adjuvant	1-year, 2-year, and 3-year survival rates; recurrence rates; quality of life; adverse reactions	Rong-rong et al. (2021)
Human	Multiple studies (systematic review)	Primary liver cancer (hepatocellular carcinoma)	Traditional Chinese medicine (including huaier granule) + radiofrequency ablation	Radiofrequency ablation alone or TCM alone	Varied	Reduce alpha-fetoprotein and improve clinical efficacy	Early to advanced HCC	1-year, 2-year, and 3-year OS; 1-year, 2-year, and 3-year RFS; complete ablation rate; liver function; adverse events	Kong et al. (2023)
Human	178 (89/89 (after PSM))	Unresectable hepatocellular carcinoma	Huaier granules + targeted therapy (sorafenib/lenvatinib/apatinib) + immunotherapy (PD-1/PD-L1 inhibitors)	Targeted therapy + immunotherapy alone	Median 12.3 months (range 3–36 months)	Prolonged PFS and improved quality of life	Advanced/unresectable HCC	OS; PFS; ORR; DCR; adverse events	Li H. et al. (2025)
Human (bioinformatics + clinical validation)	124 patients (TCGA + validation cohort)	Hepatocellular carcinoma	Huaier (a potential targeted drug)	N/A (bioinformatics study)	N/A	Improved T cell-mediated tumor killing	Progression/hyper-progression recurrence	Immune cell infiltration patterns; immune-related gene expression; huaier target prediction; survival analysis	Xu et al. (2025)
Human	60 (30/30)	Advanced triple-negative breast cancer (rescue treatment)	Huaier granules + immunotherapy (PD-1/PD-L1 inhibitors)	Immunotherapy alone	Until disease progression or intolerable toxicity	Improved T-cell immune function and prolonged PFS and OS	Advanced/metastatic TNBC (after progression on prior therapies)	ORR; DCR; PFS; OS; quality of life; immune function (CD3^+^, CD4^+^, CD8^+^, NK cells)	Yu-lai et al. (2024)
Human	Multiple studies (55 RCTs, n = 8,876 patients)	Hepatocellular carcinoma	Multiple adjuvant treatments, including huaier granule, TACE, RFA, interferon, sorafenib, lenvatinib, etc. (alone or in combination)	Placebo or no adjuvant treatment	Varied (typically 1–2 years)	Enhanced radiation-induced cytotoxicity	Postoperative (after curative resection)	1-year, 2-year, 3-year, and 5-year OS; 1-year, 2-year, 3-year, and 5-year RFS; recurrence rate; adverse events	Ye et al. (2023)

T, treatment group; C, control group; OS, overall survival; PFS, Progression-Free Survival; RFS, Recurrence-Free Survival; DFS, Disease-Free Survival; TACE, transarterial chemoembolization; RFA, radiofrequency ablation; PSM, propensity score matching; KPS, karnofsky performance status; ORR, objective response rate; DCR, disease control rate; TID, Ter In Die (three times daily); TCGA, the cancer genome atlas; TNBC, Triple-Negative Breast Cancer; HBV, Hepatitis B Virus; FEC, 5-Fluorouracil + Epirubicin + Cyclophosphamide; TC, Docetaxel + Cyclophosphamide; TAC, Docetaxel + Doxorubicin + Cyclophosphamide; TP, Docetaxel + Cisplatin; PC, Paclitaxel + Carboplatin; ECT, Epirubicin + Cyclophosphamide + Docetaxel; VE, Vinorelbine + Epirubicin; FOLFOX4, Oxaliplatin + 5-FU + Leucovorin; XELOX, Capecitabine + Oxaliplatin; ALT, alanine aminotransferase; AST, aspartate aminotransferase; TBIL, total bilirubin; ALB, albumin; CEA, carcinoembryonic antigen; CA724, Cancer Antigen 72–4; CA199, Cancer Antigen 19–9; SF-36, Short Form-36; N/A, not applicable.

**TABLE 3:** Summary of clinical studies and trials in integrated traditional Chinese and Western medicine antitumor therapy: key supporting preclinical evidence.

Species	Sample size (T/C)	Cancer type	Intervention (T)	Intervention (C)	Intervention time	Outcome	Stage	Primary endpoint	References
Mice	*In vitro*: Cell lines; In vivo: mouse xenograft model	Non-small-cell lung cancer	Huaier + cisplatin	Cisplatin alone or huaier alone	*In vitro*: 24–72 h; In vivo: 21 days	Suppressed cisplatin resistance and cancer cell stemness	Preclinical (cell and animal studies)	Cell viability; IC50 values; apoptosis rate; cell migration and invasion; tumor volume and weight in xenograft model; JNK/JUN/IL-8 signaling pathway proteins	Jin et al. (2024)
Mice	*In vitro*: Cell lines; In vivo: mouse xenograft model	Pancreatic cancer	Huaier + gemcitabine	Gemcitabine alone or huaier alone	*In vitro*: Varied; In vivo: 28 days	Reversed gemcitabine resistance	Preclinical (cell and animal studies)	Cell proliferation; colony formation; apoptosis; stem cell markers (CD133, CD44, Sox2, Oct4); tumor growth in xenograft model; FoxM1 expression	Wang Q. et al. (2024)
*In vitro*	N/A (cell lines)	Cholangiocarcinoma	Huaier + 5-Fluorouracil	5-Fluorouracil alone or huaier alone	Not specified	Exhibited a synergistic antitumor effect, which might be associated with the activation and translocation of STAT3	*In vitro* cell study	Cell proliferation, apoptosis, cell cycle distribution, migration, and invasion	Fu et al. (2019)
*In vitro*	N/A (cell lines)	Hepatocellular carcinoma (sorafenib-resistant)	Huaier granule + sorafenib	Sorafenib alone or huaier alone	Not specified (cell experiment)	Inhibited the metastasis of sorafenib-resistant HCC	*In vitro* study	Cell proliferation inhibition rate; apoptosis rate; expression of apoptosis-related proteins (Bax, Bcl-2, Caspase-3, Caspase-9); cell cycle distribution	Wang L. et al. (2024)
Mice	*In vitro*: Cell lines; In vivo: mouse xenograft model	IK6+ Ph + acute lymphoblastic leukemia	Huaier extract + imatinib	Imatinib alone or huaier alone	*In vitro*: Varied; In vivo: 21 days	Synergized with imatinib to suppress proliferation and induce apoptosis	Preclinical (cell and animal studies)	Cell proliferation inhibition; apoptosis rate; colony formation; tumor volume in xenograft model; expression of BCR-ABL and related signaling pathways	Qu et al. (2019)
Mice	*In vitro*: Cell lines; In vivo: mouse xenograft model	Hepatocellular carcinoma	Huaier + sorafenib	Sorafenib alone or huaier alone	*In vitro*: 24–48 h; In vivo: 21 days	Inhibited tumor growth and promoted autophagy	Preclinical (cell and animal studies)	Cell viability; apoptosis rate; autophagy markers (LC3-II, Beclin-1, p62); tumor volume and weight in the xenograft model; mTOR signaling pathway proteins	Zhang et al. (2022)
*In vitro*	N/A (cell lines: RAW264.7, THP-1)	N/A (immune mechanism study)	Huaier proteoglycan	PBS or control	24–48 h	Enhanced macrophage production of nitric oxide and proinflammatory cytokines	*In vitro* mechanistic study	NF-κB and MAPK signaling activation; TLR4 pathway; cytokine production (TNF-α, IL-6, IL-1β, IL-12); macrophage activation	Yang et al. (2019)
Mice	*In vitro*: Cell lines; In vivo: mouse model (CT26)	Colorectal cancer	Huaier	PBS or control	*In vitro*: Varied; In vivo: 14–21 days	Enhanced CD8^+^ T-cell infiltration and cytotoxicity while depleting regulatory T cells	Preclinical (cell and animal studies)	MHC class I expression; CD8^+^ T-cell activation; tumor growth; immune cell infiltration; immunosuppressive factors (TGF-β, IL-10, VEGF)	Chen et al. (2025)
Mice	*In vitro*: Cell lines; In vivo: mouse model (4T1)	Triple-negative breast cancer	Huaier + anti-PD-1/PD-L1 therapy	Anti-PD-1/PD-L1 alone or huaier alone	*In vitro*: Varied; In vivo: 21–28 days	Remodeling the tumor immune microenvironment and enhancing the response to chemotherapy PD-1 blockade	Preclinical (cell and animal studies)	Cancer-associated fibroblast (CAF) suppression; α-SMA, FAP expression; CD8^+^ T-cell infiltration; tumor growth; response to immunotherapy	Li et al. (2024a)
Mice	*In vitro*: Cell lines; In vivo: mouse model (Hepa1-6)	Hepatocellular carcinoma	Huaier + anti-PD-L1 antibody	Anti-PD-L1 alone or huaier alone	*In vitro*: Varied; In vivo: 21 days	Promoted CD8^+^ T-cell tumor infiltration, upregulated PD-L1 expression, and downregulated VEGFA	Preclinical (cell and animal studies)	Tumor growth; immune cell infiltration (CD8^+^ T cells, NK cells); PD-L1 expression; tumor immune microenvironment modulation; cytokine levels	Li et al. (2024b)
*In vitro*	N/A (cell lines: MCF-7, MDA-MB-231, SK-BR-3)	Breast cancer	Huaier + radiotherapy	Radiotherapy alone or huaier alone	Not specified (cell experiment)	Induced G0/G1 cell cycle arrest and impaired DNA repair mechanisms	*In vitro* study	Cell survival fraction; colony formation; apoptosis rate; cell cycle distribution; DNA damage repair; DNA-PKcs, Ku70, and Ku80 expression	Ding et al. (2016)

T, treatment group; C, control group; IC50, Half Maximal Inhibitory Concentration; HCC, hepatocellular carcinoma; PBS, Phosphate-Buffered Saline; BCR-ABL, Breakpoint Cluster Region-Abelson fusion gene; LC3-II, Microtubule-associated protein 1A/1B-light chain 3 II; p62, Sequestosome-1; mTOR, mammalian target of rapamycin; NF-κB, Nuclear Factor kappa-light-chain-enhancer of activated B cells; MAPK, Mitogen-Activated Protein Kinase; TLR4, Toll-Like Receptor 4; MHC, major histocompatibility complex; TGF-β, Transforming Growth Factor-beta; IL, interleukin; VEGF, vascular endothelial growth factor; α-SMA, alpha-Smooth Muscle Actin; FAP, fibroblast activation protein; CAF, Cancer-Associated Fibroblast; PD-1, Programmed Cell Death Protein 1; PD-L1, Programmed Death-Ligand 1; NK, cells, Natural Killer cells; DNA-PKcs, DNA-dependent Protein Kinase Catalytic Subunit; Ku70/Ku80, DNA, repair proteins; N/A, not applicable.

### 3.2 Antitumor mechanisms of HEG

Originally utilized in traditional medicine for clearing heat, detoxification, cooling blood, and hemostasis, HEG has recently garnered significant attention for its antitumor properties and has been increasingly incorporated into clinical oncology practice. Current evidence indicates that HEG exerts antitumor effects through multiple mechanisms, including induction of apoptosis, inhibition of tumor angiogenesis, immunomodulation, mitigation of oxidative stress, suppression of tumor cell proliferation, and promotion of differentiation. Although the components of Huaier granules are complex, exploring the molecular mechanisms from the perspective of constituent-mechanism analysis provides a feasible approach. Table 4 summarizes the proposed relationships between the antitumor mechanisms and their corresponding active constituents. Elucidating these mechanisms is essential for developing novel strategies for integrating HEG into combination cancer therapies.

**TABLE 4:** Proposed mechanism-constituent relationships.

Signaling pathway	Proposed constituent	Evidence level	Supporting studies	Limitations
PI3K/Akt/mTOR	TPG-1 (proteoglycan)	Moderate	Zhang 2021 (HepG2), Li 2020 (H22)	Only *in vitro* and murine models; human tumor tissue data were lacking
JAK/STAT3	PS-T (polysaccharide)	Low-moderate	Wang 2019 (A549), Chen 2022 (MCF-7)	Inconsistent results across cell lines; upstream targets unclear
Wnt/β-catenin	Unspecified polysaccharide	Low	Liu 2020 (SW480)	Single study; specific active component not identified
NF-κB/inflammation	Crude HEG extract	Moderate	Multiple studies (HepG2, H22, and clinical biomarkers)	Anti-inflammatory effects confirmed in clinical serum markers (IL-6, TNF-α↓)
Autophagy	TPG-1	Low	Chen 2021 (HepG2)	Pro- and antiautophagic effects are context-dependent
miRNA regulation	Unspecified	Very low	Single study (miR-26a in HCC)	Mechanism unclear; clinical relevance unknown

HEG, huaier granules; PI3K, Phosphatidylinositol 3-Kinase; Akt, Protein Kinase B; mTOR, mammalian target of rapamycin; TPG-1, Trametes robiniophila Proteoglycan-1; JAK, janus kinase; STAT3, Signal Transducer and Activator of Transcription 3; PS-T, polysaccharide from trametes robiniophila; Wnt, Wingless-related integration site; NF-κB, Nuclear Factor kappa-light-chain-enhancer of activated B cells; IL-6, Interleukin-6; TNF-α, Tumor Necrosis Factor-alpha; miRNA, MicroRNA; HCC, hepatocellular carcinoma; HepG2, human liver cancer cell line; H22, Murine hepatoma cell line; A549, Human lung cancer cell line; MCF-7, human breast cancer cell line; SW480, Human colorectal cancer cell line.

#### 3.2.1 Inducing cell apoptosis

Studies indicate that HEG can effectively induce tumor cell apoptosis, a key mechanism underlying its antitumor activity. It reduces tumor cell populations and inhibits tumor progression by promoting apoptotic cell death. It exhibits potent pro-apoptotic effects across various hematologic and solid tumors, causing dose- and time-dependent suppression of cell viability in T-cell acute lymphoblastic leukemia (T-ALL), lung cancer, and other malignancies (Qin et al., 2024). Mechanistically, HEG induces mitochondrial-mediated apoptosis by up-regulating cytochrome c, cleaved caspase-3, cleaved PARP, and p53, while also modulating autophagy and inhibiting survival pathways, such as SIRT1 and ATG7 (Ji et al., 2024). It further impedes tumor metastasis and proliferation by suppressing JAK2/STAT3 and MAPK signaling, inhibiting epithelial–mesenchymal transition, and altering the Bax/Bcl-2 ratio (Chen Y. et al., 2018). Additionally, Huaier extract facilitates mitochondrial apoptosis in breast cancer, melanoma, and lung adenocarcinoma by downregulating Bcl-2, up-regulating Bax and p53, activating caspase-3, and inducing cell cycle arrest. It also modulates tumor-suppressive miRNAs, including miR-26b-5p, to inhibit EZH2 and enhance pro-apoptotic signaling (Zhang et al., 2010; Zhang et al., 2013; Wu et al., 2014).

#### 3.2.2 Inhibiting tumor angiogenesis

Tumor growth and metastasis rely on angiogenesis, the formation of new blood vessels. HEG effectively inhibits tumor angiogenesis, thereby restricting the supply of blood and nutrients to tumors and suppressing their growth and metastatic potential. Huaier extract and its purified polysaccharide components, such as SP1 and TP-1, exhibit strong antiangiogenic properties in various tumor models, including hepatocellular and breast carcinomas. These effects are achieved through dose-dependent inhibition of endothelial cell proliferation, migration, and tube formation, as well as the suppression of pro-angiogenic signaling molecules, such as VEGF, HIF-1α, STAT3, and ERK (Wang et al., 2012). *In vivo*, HEG significantly reduces microvessel density (MVD) and inhibits neovascularization in models such as the chick chorioallantoic membrane and rat aortic rings (Zou et al., 2015). It also downregulates metastasis-related factors such as MMP-2, MTDH, and N-cadherin, while up-regulating pro-apoptotic proteins such as Bax, further limiting tumor vascularization and growth (Yang et al., 2015). These multitarget antiangiogenic actions, along with its broader regulation of oncogenic pathways and induction of tumor cell apoptosis, reinforce the potential of HEG as a promising antiangiogenic agent for cancer therapy (Li et al., 2015).

#### 3.2.3 Regulates immune function

HEG exerts substantial immunomodulatory effects within the tumor microenvironment and enhances systemic antitumor immunity through diverse mechanisms. As an adjuvant cancer therapy, HEG significantly improves immune parameters, including T lymphocyte subsets (CD3^+^, CD4^+^, CD4^+^/CD8^+^ ratio), immunoglobulins (IgA, IgG, and IgM), and NK cell activity (Long and Wu, 2023; Zang et al., 2024). Its broad-spectrum immunomodulatory properties target innate and adaptive immunity by regulating multiple immune cells and signaling pathways to restore immune homeostasis, demonstrating high potential for treating immune-related diseases with favorable efficacy and safety. HEG inhibits tumor growth by promoting dendritic cell maturation and shifting immune responses toward Th1 polarization via modulation of the MAPK and PI3K/Akt pathways (Pan et al., 2020). It also induces immunogenic cell death in tumor cells by activating the circCLASP1/PKR/eIF2α axis, resulting in calreticulin exposure and the release of ATP and HMGB1, which promote dendritic cell maturation and T cell activation (Li et al., 2022). Furthermore, HEG modulates the gut microbiota by enriching Akkermansia, whose metabolite butyrate enhances antitumor immunity. These immunomodulatory effects support its synergy with immune checkpoint inhibitors, helping convert immunologically “cold” tumors into “hot” tumors and providing a strong rationale for its clinical use in combination immunotherapy (Wang et al., 2025).

#### 3.2.4 Inhibiting tumor cell proliferation and promoting differentiation

HEG inhibits tumor cell proliferation while promoting differentiation toward a more normal cellular phenotype. Huaier extract exerts multitarget antitumor effects in various cancers by modulating key signaling pathways and cellular processes. In cholangiocarcinoma, it suppresses cell viability, proliferation, migration, and invasion and promotes apoptosis by downregulating the AKT/RPS6 pathway and activating the P53 pathway, as well asmodulating autophagy-related genes BECN1, ATG7, and DRAM1 (Yang et al., 2025). In gastric cancer, HEG n-butanol extract inhibits proliferation and metastasis by suppressing the c-Myc–Bmi1 axis (Wang Y. et al., 2019). In gastrointestinal stromal tumors (GIST), HEG targets the JAK2/STAT3 pathway, inhibiting proliferation and inducing apoptosis in both *in vitro* and *in vivo* models (Cao et al., 2025). In cutaneous squamous cell carcinoma (CSCC), HEG reduces DNMT1-mediated methylation of CDKN2A and TP53, thereby curbing tumor progression (Wang L. et al., 2021). Additionally, HEG aqueous extract suppresses tuberous sclerosis complex cell models by inhibiting the JAK2/STAT3 and MAPK pathways, and in NSCLC, it impedes malignancy via the lncRNA DLEU2/miR-212-5p/ELF3 axis (Yang et al., 2016; Wu et al., 2024; Hu et al., 2016; Ying-ying et al., 2020). Yang et al. reported that the proteoglycan TPG-1 exhibits potent anti-hepatoma activity by inhibiting the proliferation and migration of human SK-HEP-1 hepatoma cells and promoting apoptosis (Ai-lin et al., 2022). These findings collectively demonstrate that HEG functions via pleiotropic mechanisms—including signal transduction inhibition, epigenetic regulation, and RNA networks—underscoring its broad therapeutic potential as an anticancer agent.

#### 3.2.5 Regulating resistance to antitumor drugs through signaling pathways

**FIGURE 3:**
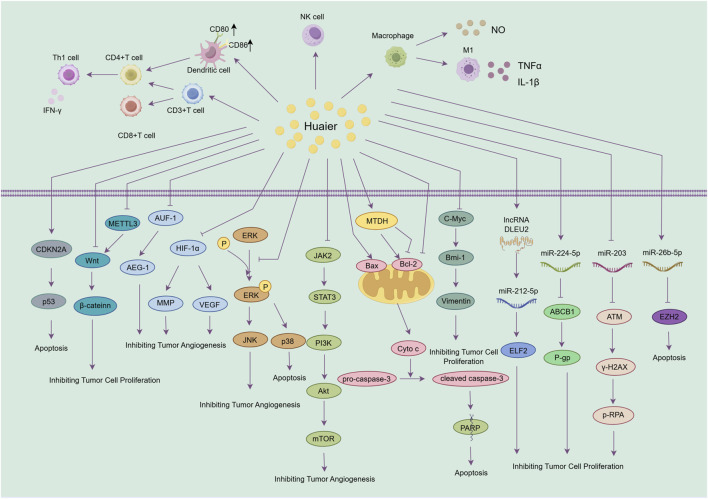
Schematic diagram illustrating the molecular mechanisms underlying the antitumor effects of Huaier granules (HEG). HEG modulates multiple signaling pathways, leading to inhibition of tumor cell proliferation, induction of apoptosis, and suppression of angiogenesis, thereby ultimately suppressing tumor progression and metastasis.

**FIGURE 4:**
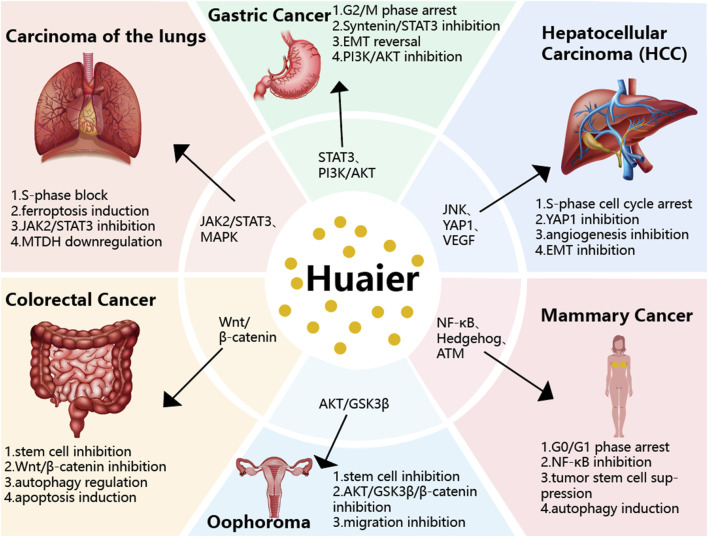
Schematic diagram illustrating the main mechanism and key signaling pathways of HEG in different tumor types.

Antitumor drug resistance occurs when tumor cells become less sensitive to therapeutic agents after prolonged exposure, resulting in reduced efficacy or treatment failure. This resistance poses a major challenge in oncology, significantly affecting patient prognosis and QoL. Current strategies to overcome resistance include combination therapies and integrated treatment approaches. HEG shows considerable promise in reversing chemoresistance across multiple cancer types through various molecular mechanisms. In colorectal cancer, it overcomes oxaliplatin resistance by inhibiting the Wnt/β-catenin pathway and modulating autophagy (Feng et al., 2024). In endocrine-resistant breast cancer, HEG inhibits proliferation by inducing G0/G1 cell cycle arrest via miR-203 downregulation and ATM upregulation (Gao et al., 2017). Regarding oxaliplatin resistance in colorectal cancer and HCC, HEG targets several pathways: it downregulates METTL3 to inhibit Wnt/β-catenin signaling, suppresses YAP to promote apoptosis, and, through its polysaccharide component PS-T, enhances chemosensitivity via the miR-224-5p/ABCB1/P-gp axis (Huo et al., 2025; Tao et al., 2018; Gou et al., 2022). These results collectively establish HEG as a multitarget agent capable of reversing various chemoresistance mechanisms. Figure 3 clearly illustrates and classifies the molecular pathways involved in Huaier’s antitumor mechanisms described above. Figure 4 illustrates the main mechanisms and key signaling pathways of HEG across different tumor types, highlighting its cancer-type-specific antitumor effects.

## 4 Conclusion and discussion

### 4.1 Summary of the main findings

This systematic review of 56 studies involving 4,832 patients provides moderate-certainty evidence that Huaier granules, as adjuvant therapy, improve RFS in HCC following curative resection or ablation (HR ∼0.67). Benefits in OS are supported by lower-certainty evidence, with consistent signals across multiple cancer types. HEG appears safe, showing no increase in serious adverse events and possibly offering protective effects against chemotherapy-induced myelosuppression.

### 4.2 Conclusion

HEG demonstrate adjuvant efficacy in HCC with moderate-certainty evidence, particularly in reducing recurrence after curative treatment. The evidence base, though promising, is limited by methodological shortcomings, including unavoidable performance bias in open-label trials, heterogeneity in outcome definitions, and a disconnect between clinical formulations and mechanistic study materials. The translation of preclinical findings (immunomodulation, anti-angiogenesis, and apoptosis induction) to clinical practice remains uncertain due to the lack of pharmacokinetic bridging data and the absence of pharmacodynamic biomarkers in clinical trials. Future research must prioritize rigorous, multicenter, blinded RCTs with standardized endpoints, mechanistic substudies, and quality control measures to fully realize the potential of this traditional medicine in modern oncology practice.

### 4.3 Methodological limitations and risk of bias

#### 4.3.1 Performance bias (inevitable but impactful)

All RCTs were open-label because of the distinct appearance and taste of the HEG. While objective endpoints (OS, PFS, and recurrence) are less susceptible to bias, QoL and performance status ratings may be inflated. The magnitude of this bias is uncertain—TCM studies often report larger effect sizes than blinded Western medicine trials, suggesting possible placebo/expectation effects.

#### 4.3.2 Selection bias in observational studies

Retrospective studies likely selected patients with better performance status and fewer comorbidities for HEG treatment, despite statistical adjustments. The “healthy user” effect might exaggerate benefits.

#### 4.3.3 Heterogeneity

Despite consistent direction, effect sizes varied due to variable HEG dosing (though most used 20 g tid), different cancer stages and prior treatments, inconsistent outcome definitions (e.g., RFS vs. DFS), and varying follow-up durations.

### 4.4 Limitations of this study

One limitation of this study was the language restriction, which included only studies in English and Chinese, potentially excluding those in other languages (although HEG is primarily used in China). Second, excluding conference abstracts may have introduced publication bias. Third, variable definitions precluded meta-analysis for some outcomes. Fourth, median follow-up duration was 2–3 years in most studies, making long-term benefits (>5 years) uncertain. Finally, generalizability was limited, as the population was predominantly Chinese Han, with applicability to other ethnicities unknown.

### 4.5 Safety profile

Across the included studies, HEG demonstrated a favorable safety profile. The most reported adverse events were mild gastrointestinal discomfort (e.g., nausea, diarrhea) and rare cases of rash, with no significant increases in grade 3–4 toxicities compared to control groups. Notably, several studies reported reduced chemotherapy-induced myelosuppression in HEG combination groups (Xie et al., 2022). In immunotherapy combinations, current data do not suggest that HEG increases the frequency or severity of immune-related adverse events. However, we acknowledge a critical limitation: no pharmacokinetic studies have investigated potential drug-drug interactions between HEG and concurrently administered antitumor agents. This represents an important direction for future research.

### 4.6 Implications for future research

Further investigation into the mechanisms is essential to maximize its antitumor potential. This will aid in clarifying its pharmacological profile and strengthening the scientific foundation for its clinical application. Future research should focus on the following directions (Siegel et al., 2024): identifying the active components of HEG and elucidating their specific antitumor mechanisms (Wang et al., 2023); investigating interactions between HEG and other antitumor drugs to discover novel combination regimens (Qin et al., 2022); conducting rigorous pharmacokinetic and pharmacodynamic studies to evaluate potential drug-drug interactions between HEG and concurrently administered antitumor agents, particularly targeted therapies and immunotherapies, to ensure safe co-administration (La Torre et al., 2023); studying the relationship between HEG’s mechanism of action, patients’ genetic backgrounds, and tumor molecular typing to achieve personalized treatment; and (Kiri and Ryba, 2024) exploring the role and function of HEG in comprehensive tumor treatment to improve overall efficacy.

In summary, although the mechanisms of HEG require further in-depth study, it holds great potential for clinical antitumor therapy. Elucidating these mechanisms will provide new ideas and strategies for tumor treatment and is expected to improve patients’ QoL and survival rates.
